# Evaluating a Targeted Language Intervention for Children Aged 4–6 Years—Applying an ‘Information Carrying Words’ Approach

**DOI:** 10.1111/1460-6984.70047

**Published:** 2025-05-12

**Authors:** Sarah Spencer, Laura Pearce, Karlene Calder, Alice Woods, Abigail Andrews, Judy Clegg

**Affiliations:** ^1^ School of Allied Health Professions, Nursing and Midwifery University of Sheffield Sheffield UK; ^2^ Sheffield Children's NHS Foundation Trust Sheffield UK; ^3^ Nottinghamshire County Council Nottingham UK

**Keywords:** intervention, Language, school‐age children

## Abstract

**Background:**

Very little research has investigated the use of the information carrying words (ICW) construct within language interventions, despite its very widespread use in speech and language therapy in the United Kingdom. The Language Enrichment Activity Programme (LEAP) is an intervention programme that applies the ICW construct to differentiate children's level of need and structure play‐based learning activities. LEAP sessions are designed to be child‐led, building language skills through application of communication supporting strategies (CSS) such as modelling, recasting and inviting communicative participation via choices and expectant pausing.

**Aims:**

This study aims to evaluate the impact of trainee speech and language therapists (SLTs) delivering LEAP on the language skills of primary school children (aged 4–6 years).

**Methods and Procedures:**

One hundred eighteen children were selected by their teachers. Participating children were semi‐randomly allocated to either a control group (*n* = 48) or to a group that received 12 sessions of LEAP over 6 weeks (*n* = 70). A smaller sub‐cohort was followed up 8 weeks following the end of LEAP (received LEAP *n* = 41, control group *n* = 46). LEAP was delivered by trainee SLTs to small groups of children. Assessments were carried out blind to group allocation pre‐ and post‐intervention in order to evaluate the impact of intervention on receptive and expressive language skills. Outcome measures were a bespoke comprehension and expression outcome measure and the Renfrew Action Picture Test (RAPT; Renfrew 2019).

**Results:**

Children who received LEAP had improved scores on both the RAPT assessment and the bespoke outcome measure. There was a significant interaction between time (pre‐ and post‐intervention) and group (those who received LEAP vs. the control) for the RAPT grammar score, and the LEAP vocabulary and expressive ICW score. LEAP had less of an impact for a sub‐cohort followed up 8 weeks following the intervention, with only the LEAP expressive score showing significant interaction between the three assessment time points and group (those who received LEAP vs. the control).

**Conclusions and Implications:**

The LEAP was successfully delivered to small groups of children and supported them in developing their expressive language skills. Working with trainee SLTs increased the capacity to deliver LEAP at a low cost to schools. Results are promising and add to an emerging evidence base for the application of the ICW construct within SLT intervention programmes.

**WHAT THIS PAPER ADDS:**

*What is already known on this subject*

- Use of an information carrying words (ICW) construct in speech and language therapy is very common in the United Kingdom, and yet very little research has been done in this area. There is strong evidence for the role of communication supporting strategies (e.g., recasting, giving choices, and use of multisensory input) to accelerate children's language development. However, more translational research is needed to investigate the impact of interventions which include such strategies. New service delivery models are also needed to facilitate increased access to interventions, particularly beyond the preschool years and where schools are unable to staff comprehensive language interventions internally.

*What this study adds*

- This project evaluates the impact of a targeted language intervention programme for children in the early stages of primary school in the United Kingdom: the Language Enrichment Activities Programme (LEAP). This programme uses ICW to differentiate language intervention activities, while using robust communication supporting strategies during sessions. The study evaluates the impact of LEAP on a cohort of 118 children from 8 schools, with promising results. Children who received LEAP had increased scores on measures of expressive language when compared to children who did not take part in LEAP.

*What are the clinical implications of this study?*

- Our study adds to an emerging evidence base supporting the application of the ICW construct within language interventions. It also offers a model for collaboration between schools, speech and language therapy services, and trainee speech and language therapists to increase capacity for targeted small group interventions for language skills in early primary school. The paper uses the LEAP intervention as a starting point for discussion about how to best deliver targeted small group interventions and issues in making these types of groups inclusive and accessible.

## 1 Introduction

Speech and language therapists frequently design and deliver intervention for children's early language skills using the concept of ‘information carrying words’ (ICW), as borrowed from the Derbyshire Language Scheme (Masidlover 1979). However, very little research has examined the impact of incorporating ICW levels into differentiated language interventions. There is more evidence for the use of communication supporting strategies (CSS)—adult interaction features that facilitate communication development in children (Rowe and Snow 2020). Such strategies include commenting on what the child is doing; repeating and extending child utterances with additional semantic, syntactic, and/or phonological information (conversational recasting); and using expectant pauses and giving children choices to invite communicative participation. This project evaluates the impact of a targeted language intervention programme for children in the early stages of primary school in the United Kingdom: the Language Enrichment Activities Programme (LEAP). LEAP was designed by the Sheffield Children's NHS Foundation Trust and has formed part of the services’ offer for training for schools for many years (Sheffield Children's NHS Foundation Trust, n.d.). LEAP uses informal assessment to differentiate children's expressive and receptive language skills in terms of ICW. Children then receive a 12‐session intervention programme in small groups, with opportunities for exposure to frequent CSS. LEAP is designed to be delivered by trained school staff, typically teaching assistants and learning support assistants. However, in recent years local schools have reported challenges in staffing LEAP groups internally. This project is a provisional evaluation of the impact of working with trainee speech and language therapists to deliver LEAP.

### 1.1 ICW

Speech and language therapists in the United Kingdom very commonly base comprehension intervention activities on activities where children are asked to follow structured, play based directions with variation in the amount and variety of words that carry meaning in a sentence (‘information carrying words’) within these activities (Morgan et al. 2019). ICWs refers to the number of words within an utterance that need to be understood in order to functionally follow an instruction (or to give an instruction, if being applied expressively). Calculating the number of ICWs in an utterance does not mean simply counting the words involved. For example, the instruction ‘please can you go and put your shoes on’ is made up of nine words but could be functionally understood if a child understands just one information carrying word: shoes (or perhaps could be followed without understanding any language, if the child relies on contextual cues and routines such as an adult putting on their own shoes, with previous experience of the child putting on their shoes independently).

Context is central to an ICW approach: in each instruction, some of the utterances are made redundant by the contextual information available to the child, that is, given the context, only the words that a child must understand in order to respond correctly are considered to be the ICWs. For example, if a child is offered a bowl of sweets, with the question ‘Would you like a sweet?’ the child may take one even without understanding any of the language involved, instead relying on familiarity, routine and context. In this situation the instruction would be categorized as having no ICWs. However, if the child is asked the same question and is offered a plate containing cakes, biscuits and sweets, the child presumably needs to understand the word ‘sweet’ to follow the instruction (unless by chance they prefer sweets!). Therefore, depending on the context, the same instruction could require the child to understand a different number of ICWs. To give a further example, ‘give the teddy an apple’ would require understanding of two ICWs if there was a teddy and a doll, plus an apple and a banana. However, if there was just a teddy and the fruit, it would require an understanding of just one ICW.

ICWs have been used to differentiate children's language levels by speech and language therapists for many years (Masidlover 1979). The rationale for this is perhaps based on socio‐cultural and social‐interactionist theoretical perspectives of the time (Bruner 1983; Vygotsky 1978), which placed importance on children's early social‐communicative interactions to support language development. Vygotsky's concept of the Zone of Proximal Development suggests that the value of any intervention activity depends on the current language ability of the child; input must be challenging, but not too difficult, in order to facilitate the most progress. The child is not just a passive recipient of CSS but plays an active role in how interactions unfold. Bloom's intentionality model of language acquisition was influenced by Vygotsky (1978) and Bruner's (1983) concept of scaffolding. Bloom puts forward that children learn and use words as a result of interactions with other people, driven by the need for expression and interpretation (Bloom 2000), principles that fit well with the use of the ICW construct within language interventions.

Very little research has examined the ICW construct in assessment or intervention, with some exceptions (Anderson 2006). A study of 27 children found that children's receptive language skills, as conceptualised by the number of ICWs that they could understand in instructions, were strongly correlated with performance on broader expressive and receptive language measures (Frizelle et al. 2017). This adds some early empirical evidence to the common clinical practice of using ICW as a proxy for overall language level, although there were also strong associations with working memory. Current research is underway to examine the impact of interventions applying an ICW construct for preschool aged children as part of Elkan's Early Years Based Information Carrying Word Programme (Clegg et al. 2023) and in a study comparing the Building Early Sentences Therapy approach to an adapted Derbyshire Language Scheme, again for preschool children (McKean et al. 2023). Early results suggest that the ICW approach has some value with younger children. This study evaluates the application of the ICW construct in early primary school to differentiate and structure an intervention based on CSS.

### 1.2 CSS

There is a robust and growing evidence base for the use of CSS—also referred to as focused stimulation, communication‐facilitating behaviours, or language‐facilitating techniques or interactions—to support children's early language development (Adamson et al. 2020; Justice et al. 2018). Such strategies include modelling, elicited production, and recasting. For example, during conversational recasting an adult responds to a child's utterance with a repetition or partial repetition, adding new language while maintaining the same basic meaning expressed by the child (see Cleave et al. 2015, for a review).

In this study, the CSS that formed part of LEAP included: (1) warm manner and looking expectantly to encourage communicative participation; (2) maintaining a slow pace to give children time to participate; (3) eliciting child contributions using expectant pauses and extending contributions using open‐ended questions; (4) encouraging and supporting talk among peers (Justice et al. 2018); (5) recasting child contributions during activities; (6) responding contingently, focusing on the child's actions and spoken contributions in that moment; and (7) modelling and repetition of key vocabulary and utterances in relation to the targeted ICW level.

LEAP uses play activities that are structured in a way that allows frequent use of CSS naturally within interactions. These strategies are embedded in a naturalistic and interactive context, with opportunities for the children to take the lead and to learn from each other. The trainee SLT is given a session plan, based on the ‘level’ of ICW that a child is currently at, and responds flexibly to a child's verbal contributions and nonverbal ways of participating in the session.

### 1.3 Targeted Interventions

LEAP is designed by SLTs as a Targeted programme (also referred to as ‘Wave 2’ or ‘Tier 2’), to be delivered by education staff to children in small groups. Such interventions are typically offered to children who do not necessarily have a diagnosis of a developmental language disorder, but who nevertheless have language skills that currently disadvantage them in the classroom (Ebbels et al. 2019). Such intervention approaches are common in preschools, with a variety of manualised programmes available (Clegg et al. 2020; Fricke et al. 2013; Lonigan and Phillips 2016; Reeves et al. 2018; Trebacz et al. 2024; Walker et al. 2020). Fewer targeted intervention programmes are available for primary school aged children, though a very well‐known and well‐evaluated exception is the Nuffield Early Language Programme (NELI). Randomised control trials have established the efficacy and cost‐effectiveness of NELI (Dimova et al. 2020; Fricke et al. 2017). Targeted intervention packages are typically purchased with a cost to schools, for initial ‘buy in’ of resources and staff training, plus the investment of staff time to run the interventions. For example, NELI initially costs £870 for materials for a single form entry school (Education Endowment Foundation 2020), and then schools’ teaching assistants run the programme, at a cost of approximately £3500 per eight participating children (Snowling et al. 2022). NELI is delivered over 20 weeks, with two 15‐min individual sessions and three 30‐min small group sessions per week for each child. Although many school leaders and teachers report that this is a worthwhile investment (Snowling et al. 2022), it is likely that some schools do not have the capacity to deliver NELI without additional staffing, particularly in a context of worsening staff shortages (Ofsted 2022).

### 1.4 The Current Study

Use of the ICW construct in SLT is very common in the United Kingdom (Roulstone et al. 2012) and yet has a limited evidence base, with very little research evaluating interventions that differentiate the level of support based on the number of ICW that children can understand or produce. While there is strong evidence for the role of CSS to accelerate children's language development, more research is needed to investigate how to facilitate children's access to interventions that feature CSSs, particularly beyond the preschool years. There is a robust evidence base for the school‐delivered targeted language intervention NELI. Where schools are unable to staff comprehensive programmes such as NELI, there is very little research into alternative service delivery models for running targeted Tier 2 language interventions to support the transition to school. This project evaluates the impact of LEAP—a relatively short, targeted intervention for children in the first 2 years of school (4 to 6 years). We worked with trainee SLTs to deliver LEAP, to have the dual benefit of providing clinical placement opportunities for the trainees and increasing schools’ capacity to run language interventions. Our project addressed the research question: What is the impact of LEAP on children's spoken language skills?

## 2 Method

### 2.1 Ethics

The project was granted ethical approval by the University of Sheffield's Department of Human Communication Sciences Ethical Committee. Informed consent was obtained from headteachers, teachers and parents/caregivers before participation.

### 2.2 Study Design

The study has a quasi‐experimental design, with a waiting control and pre‐ and post‐test design. We recruited children from eight schools in Sheffield, South Yorkshire, UK, in partnership with the SLT team in the Sheffield Children's National Health Service (NHS) Foundation Trust. The characteristics of these schools are summarised in Table 1. All eight schools had a much higher proportion of children who were entitled to free school meals (FSM), an indicator of low household income in the United Kingdom, when compared to national average rates of entitlement. Using information in the latest Ofsted reports for each school, five schools had a catchment that was mostly White British, and three had a majority of pupils that were from a minority ethnic background, with a high proportion speaking a language other than English at home.

**TABLE 1:** Characteristics of the eight participating schools based on most recent Ofsted report.

School	Proportion of children in receipt of free school meals	Proportion of children with English as an additional language	Ethnicity data as described by Ofsted	Special Educational Needs (SEN) data	Number of enrolled children
1	54%	Well above average	The majority of pupils are from the Pakistani ethnic group. There are increasing numbers of pupils from Gypsy, Roma and Eastern European groups joining the school.	Above average	400+
2	47%	Below average	Majority White British ethnicity.	Around average	200+
3	63%	Well above average	Almost all pupils are from minority ethnic backgrounds, mainly Asian ethnic groups.	Around average	400+
4	49%	Below average	Majority White British ethnicity	Around average	300+
5	52%	Below average	Majority White British ethnicity.	Around average	700+
6	53%	Below average	Majority White British ethnicity.	Around average	700+
7	41%	Above average	Almost all pupils are from minority ethnic backgrounds. A high and increasing number of pupils are from a Roma ethnic group or are Slovakian.	Above average	200+
8	38%	Below average	Majority White British ethnicity.	Well above average	300+
Average (England)	24%	21%	NA	NA	280

The SLT team had developed the LEAP programme many years ago, and it was an established targeted Tier 2 intervention in the area, with the NHS team offering training for school staff in order for them to deliver the programme independently. This project explored the impact of working with trainee SLTs to deliver LEAP and gathered provisional evaluation data for LEAP.

Participating children were in reception or Year 1 (4–6 years old). The information for schools stated that Year 2 children could participate, but of the small number of Year 2 children identified by teachers, none met our inclusion criteria. All the children were assessed before intervention to establish eligibility for the study and to act as a baseline, with assessments repeated as outcome measures following 6 weeks of twice weekly intervention. In the original design, the waiting control group was scheduled to then receive the LEAP intervention, but this was not possible due to school closures in 2020 during the global pandemic. We also looked at changes for a smaller cohort of 80 whom we followed up approximately 8 weeks after they completed the LEAP programme (for 41 children who had completed LEAP and 46 who had not completed LEAP). Again, this smaller cohort was due to school closures. The children with only Time 1 and Time 2 data completed LEAP in the period of time between school closures. Children with Time 3 data completed all three assessments before school closures.

### 2.3 Participants

Participating children were selected by their teachers based on inclusive guidelines, as outlined below.

Teachers were asked to identify children who:

- Had spoken language abilities notably behind their peers.
- Have some spoken English language skills (e.g., use of some single words).
- Were in one of the first three classes of primary school, referred to in England as Key Stage 1: Foundation Stage Two (Reception), Year 1 and Year 2 groups.
- May or may not have English as Additional Language (EAL): this was not an exclusion criterion.
- May or may not have a diagnosis leading to special educational needs (SEN): this was not an exclusion criterion.
- May or may not have had previous contact with a speech and language therapist: this was not an exclusion criterion.

Guidelines were provided to help teachers identify children who had spoken language abilities notably behind their peers. If the child was bilingual or multilingual, we advised teachers that there should be concern about the first language as well as English (as discussed with bilingual teaching staff and/or families). Teachers then contacted these children's families and invited them to take part in the LEAP group and the evaluation research project (families were welcome to take part in LEAP without being part of the assessment for the evaluation, and four families opted to do so). Teachers reported that no families decided not to take part in the intervention. Following the completion of parental consent forms, the recruited children were then assessed by the trainee SLTs with support from supervising SLTs and the lead author (see below for assessments used).

In total, 159 children were recruited for the evaluation study, though 19 children were excluded as assessment showed that the intervention was not suitable for their needs (language was in line with expected development) and 11 were excluded as they moved schools during the project, leaving 129. Eleven children were absent from school during the Time 2 assessment period. Therefore, 118 children participated in the evaluation study: 70 were assigned to the LEAP intervention condition, and 48 to the waiting control condition.

### 2.4 Materials

#### 2.4.1 Assessments

There are two main assessments used pre‐ (Time 1) and post‐intervention (Time 2), with a smaller sub‐cohort being assessed again approximately 8 weeks following the end of the intervention (Time 3).

#### LEAP non‐standardised assessment

The LEAP assessment is a non‐standardised measure of receptive and expressive language abilities that links directly to the intervention. It was developed by the SLT team in the Sheffield Children's NHS Foundation Trust and had been used for many years to assign children to LEAP differentiated groups. There are three subtests:

1. LEAP Vocabulary checklist (LEAP Vocab). The child is presented with a series of 50 pictures and is asked to name the target word for each picture. Items are grouped into categories such as body parts, animals, action words and foods. This section is scored out of 50.
2. LEAP ICW Receptive Language. This is an informal assessment, where children are asked to listen to a series of instructions and then respond by manipulating objects, toys and pictures. The instructions become progressively more complex, moving from 2 ICW to 4 ICW. For example, 2 ICW instructions include ‘make teddy jump’ (with a teddy and doll and modelling of other actions prior to the direction) and ‘give teddy the carrot’ (with a doll, teddy, carrot and apple on the table); 3 ICW instructions include ‘give the big carrot to dolly’ (with a doll, teddy, big and small carrots and big and small apples on the table); 4 ICW instructions include ‘put the big apple under the plate’ (with a doll, teddy, cup, plate, big and small carrots and big and small apples on the table). There were three items for each of the three ICW levels, so scores are out of 9.
3. LEAP ICW Expressive Language. The same principles are then applied to the expressive language section of the LEAP assessment. The adult performs an action, and the child is asked to describe what is happening, and as above, the context is controlled for ICW level. Targets are marked correctly if they involve the targeted number of ICW (e.g., dolly sleeping for 2 ICW (irrespective of whether they say doll/dolly/baby), teddy eating a little carrot for 3 ICW, the little banana is in the cup for 4 ICW). There were three items for each of the three ICW levels, so scores are out of 9.

#### The Renfrew Action Picture Test (RAPT)

The RAPT (Renfrew 2019) is a standardised measure of expressive language for children aged 3–8 years. It involves answering questions about 10 picture prompts, such as ‘What is the big girl doing?’ Responses are transcribed and scored for both grammatical complexity and information content, with raw scores and age‐based percentile rank scores.

### 2.5 Intervention

The LEAP is manualised and includes an interaction strategy guideline, weekly group session plans, printed and laminated pictures, and resources including teddy bears, small toy animals and vehicles, toy plates and cups, plastic food, doll and dolls clothing. LEAP covers six themes (at home, transport, actions, clothes, animals and body parts), with two sessions focusing on each theme. The LEAP programme provides session plans for each theme targeting differentiated levels—2, 3 and 4 ICW levels—with detailed guidance on how to tailor the resources to meet the language levels of individual children.

The intervention is focused on group activities as opportunities for the trainee SLTs to apply communication supporting interaction strategies throughout. For example, activities were used as an opportunity for:

- Commenting on what was happening in the activities
- Asking open questions
- Giving choices as a scaffolded means of participating, either verbally or by looking or pointing
- Repeating unknown vocabulary
- Modelling utterances at ‘level up’ from the child's current level of expressive language,
- Contingent responses to child vocalisations/utterances
- Recasting child utterances
- Slow pace of communication
- Facilitating turn taking and listening to other children as well as adults
- Using expectant pauses/sentence completion and pauses.

Children are encouraged to join in, either verbally or by pointing or using gestures. The programme is designed to boost children's skills and confidence in joining in the small group activities, without making any ‘errors,’ as gradual prompts and scaffolds are used to differentiate invited participation. All intervention activities were delivered in English. When working with bilingual children, students were encouraged to be curious about children's first language, for example, by inviting children to teach the adults words from their first language during the activities. Students were asked to follow the LEAP session plans but to work on the principle that any input from an adult needed to be in line with and responsive to the child's needs, abilities and interests (Rowe and Snow 2020). Therefore, there was some flexibility in how LEAP sessions could be delivered, building on the expertise of the trainee SLTs.

### 2.6 Procedure

Trainee SLTs were involved in the assessment of the children. The trainees were first trained in assessment, with the first author demonstrating the assessments in university workshops and then supervising clinicians demonstrating, observing and providing feedback in relation to assessments in school.

All assessment sessions took place with individual children within quiet rooms in the children's school. The first assessment session was before the intervention phase (Time 1). Assessment sessions took approximately 20 min.

While there was no reliability data available for our assessments, all trainee speech and language therapists discussed their assessment scoring with their allocated supervising clinician, and approximately half of all assessments were reviewed and discussed by two or more trainee or qualified speech and language therapists during our placement workshops. Any issues or discrepancies in scoring were discussed and resolved.

Following the Time 1 assessments, trainee SLTs worked with the lead author to allocate children to either the group to receive LEAP intervention immediately or the control group, originally planned to be a waiting control, with LEAP delivered 8 weeks later. School closures prevented this. Allocation was semi‐random, in that participants were first categorized into strata based on school, year group, and ICW assessment data (e.g., 3 ICW level or 4 ICW), and then randomly assigned to either receive intervention or be in a waiting control (so that intervention ran in each school). Random allocation was not possible because a LEAP intervention group ran in each school, and the groups were organised so that children in the same year and children who were working on similar targets were together in groups. No child had a receptive score of 1 ICW at the Time 1 assessment. Children were grouped to receive the LEAP at either 3 ICW (if they were currently functioning at 2 ICW receptively) or 4 ICW level (if they were currently functioning at 3 ICW receptively). This meant that activities and games included instructions and invitations to ‘be the teacher’ and give instructions at either 3 or 4 ICW levels. Each group had between 3 and 5 children.

Regarding the LEAP intervention, trainee speech and language therapy students first received 8 h of training (four 2‐h training sessions) with the first author, covering use of communication strategies, information carrying word principles, and guidance on how to deliver the LEAP sessions. They then had tutorial support provided by both a) the clinical placement coordinator (the first author) and b) the NHS therapist working with the school partner. The tutors visited students up to four times to observe, give feedback on use of CSS and session management, and to ensure fidelity in delivering the sessions (typically once or twice by the supervising clinical academic and twice by the NHS SLT partnered with the school). Tutors used an observation table to tally the frequency of CSS used by the students with columns for: slow pace; use of gesture and visuals; recasting child utterances with an expansion or remodelling; inviting participation with expectant pauses; inviting participation with choices; and giving first sound cues as prompts. Feedback was also provided on: (a) the students’ ability to relate to children warmly; (b) the students’ ability to differentiate both instructions and feedback depending on an ICW construct and knowledge of the child's current level of language ability; (c) where applicable, how well students built on bilingual children's first language during the sessions.

LEAP intervention was delivered by two trainee SLT students in each school twice a week over a school half‐term. The 12 sessions lasted approximately 30–45 min each, with LEAP involving around 9 hours of intervention in total.

Following intervention, assessments of the children were carried out again (referred to as Time 2). To guard against expectancy bias, the trainee SLTs swapped schools to collect Time 2 and Time 3 assessments and were blind as to whether the child had received LEAP or not. As the trainee SLTs were randomly allocated to schools, they were therefore an unfamiliar adult to the children. The LEAP and RAPT assessments for all the children were carried out within 1 week of their last intervention session (Time 2). We also looked at any changes for a smaller sub‐cohort of 80 who followed up approximately 8 weeks after the completion of the LEAP programme (41 children who had completed LEAP and 46 who had not completed LEAP) (Time 3). This was a smaller cohort due to school closures.

## 3 Analysis

Data were entered into SPSS version 29. Descriptive and statistical analysis was then completed. The mean scores, standard deviations (SD) and ranges are given for the control and LEAP groups. A two‐factor mixed‐design ANOVA was conducted for LEAP Receptive Language, LEAP Vocabulary, LEAP Expressive Language, RAPT Grammar and RAPT Information raw scores. The purpose of this ANOVA analysis was to determine the interaction between time and group, a main effect of time and a main effect of group. Post hoc *t*‐tests were then used to statistically analyse any change in scores from Time 1 to Time 2 to Time 3 for the control and LEAP groups.

## 4 Results

Participant characteristics are shown in Table 2. There were no differences in children's age, gender, or year group; the difference in the proportion of EAL children did not reach significance (chi‐square (*χ* ^2^(1) = 2.86, *p* = 0.091).

**TABLE 2:** Characteristics of participants by group at Time 1.

		Intervention group (*n* = 70)	Control group (*n* = 48)
**Age in months *M* (SD)**		60 (7)	60 (6)
**Gender**		36 (51.4): 34 (48.6)	24 (50): 24 (50)
**Girls: boys (%)**			
**EAL (%)**		28 (40)	12 (25)
**School year group**	**Reception (%)**	52 (74.3)	39 (81.3)
	**Year 1 (%)**	18 (25.7)	9 (18.8)

Table 3 shows the performance at Time 1 (pre‐intervention) and Time 2 (post‐intervention for those who received LEAP) by group for all five language measures. At Time 1, independent *t*‐tests indicated that there were no significant differences between the intervention and control groups for the LEAP vocabulary score (*t*(116) = −1.787, *p* = 0.067); LEAP ICW receptive language (*t*(116) = −1.181, *p* = 0.240); the LEAP ICW expressive language (*t*(116) = −1.386, *p* = 0.169); nor the RAPT information raw score (*t*(116) = −1.556, *p* = 0.122). However, there were significant differences between groups at Time 1 for RAPT information standard score (*t*(116) = −2.195, *p* = 0.030) *d* = 0.41, 95% confidence interval for the difference between the means was 0.78 to 0.39); RAPT grammar raw score (*t*(116) = −2.249, *p* = 0.026, *d* = 0.42, 95% confidence interval for the difference between the means was 0.79 to .049); RAPT grammar standard score (*t*(116) = −2.2821, *p* = 0.003, *d* = 0.53, 95% confidence interval for the difference between the means was 0.90 to 0.15).

**TABLE 3:** Performance at Time 1 and Time 2 on outcome measures by group.

	Intervention group *N* = 70			Control group *N* = 48		
	Time 1	Time 2		Time 1	Time 2	
	*M* (SD)	*M* (SD)	Mean change to mean score	*M* (SD)	*M* (SD)	Mean change to mean score
**LEAP vocabulary**	38.43 (11.00)	42.64 (10.10)	4.21	41.85 (9.05)	44.42 (7.96)	2.57
**LEAP ICW expressive language**	1.96 (1.89)	3.89 (2.61)	1.93	2.48 (2.17)	3.40 (2.36)	0.92
**LEAP ICW receptive language**	6.32 (2.83)	7.44 (1.98)	1.12	6.92 (2.37)	7.60 (2.00)	0.68
**RAPT grammar raw score**	10.47 (7.27)	14.00 (7.18)	3.53	13.49 (6.98)	14.80 (6.46)	1.31
**RAPT grammar percentile**	5.52 (9.48)	12.10 (18.42)	6.58	14.23 (23.03)	13.31 (17.39)	−0.92
**RAPT information raw score**	17.02 (8.46)	22.00 (8.85)	4.98	19.33 (7.07)	22.39 (8.13)	3.06
**RAPT information percentile**	10.91 (14.98)	25.86 (25.37)	14.95	17.48 (17.28)	28.48 (27.58)	11.00

Analysis then evaluated the impact of LEAP on language outcomes for both groups. The statistical analysis found no significant interaction between group (control vs. LEAP) and time (Time 1 and Time 2) for the LEAP ICW Receptive Language (*F*(1, 116) = 1.115, *p* = 0.293, partial eta squared 0.01); or the RAPT information score (*F*(1, 116) = 0.555, *p* = 0.458, partial eta squared 0.005). However, there were significant interactions between group and time for the RAPT grammar raw score (*F*(1, 116) = 4.10, *p* = 0.05, partial eta squared 0.034); the LEAP vocabulary (*F*(1, 116) = 4.346, *p* = 0.039, partial eta squared 0.036); and the LEAP ICW expressive language (*F*(1, 116) = 4.15, *p* = 0.04, partial eta squared 0.035).

Post hoc independent *t*‐tests were then conducted on the progress made between Time 1 and Time 2 by group. Results indicate progress on RAPT Grammar raw scores was higher for children who received LEAP (mean = 3.52, SD = 5.99) than those who did not receive LEAP (mean = 1.31, SD = 5.61) (*t*(116) = 2.026, *p* = 0.023). LEAP vocabulary progress was higher for children who received LEAP (mean = 4.21, SD = 4.27) than those who did not receive LEAP (mean = 2.56, SD = 4.16) (*t*(116) = 2.085, *p* = 0.020). LEAP ICW Expressive Language progress was higher for children who received LEAP (mean = 1.93, SD = 2.61) than those who did not receive LEAP (mean = 0.92, SD = 2.71) (*t*(116) = 2.038, *p* = 0.022).

Table 4 shows the characteristics of the sub‐cohort of participants who also completed follow‐up assessments (Time 3) compared to the participants with only Time 1 and Time 2 data. Independent *t*‐tests showed that the sub‐cohort with Time 3 data had higher scores for: LEAP Vocabulary *t*(116) = −2.69, *p* = 0.010, *d* = 0.66, 95% confidence interval for the difference between the means was 11.54 to 1.63; LEAP ICW receptive language *t*(116) = −2.20, *p* = 0.029, *d* = 0.46, 95% confidence interval for the difference between the means was 2.29 to 0.12; RAPT grammar raw score *t*(116) = −3.76, *p* ≤ 0.001, *d* = 0.79, 95% confidence interval 8.29 to 2.52; RAPT grammar percentile *t*(116) = −3.76, *p* ≤ 0.001, *d* = 0.53, 95% confidence interval 13.20 to 4.26; RAPT information raw score *t*(116) = −3.37, *p* = 0.001, *d* = 0.71, 95% confidence interval 8.56 to 2.38; RAPT information percentile *t*(116) = −3.44, *p* ≤ 0.001, *d* = 0.61, 95% confidence interval 15.04 to 4.01. There were no differences between the cohorts on LEAP ICW expressive language *t*(116) = −1.03, *p* = 0.308, *d* = 0.21, 95% confidence interval 1.19 to 0.38.

**TABLE 4:** Characteristics of the sub‐cohort of participants who also completed follow‐up assessments (Time 3) compared to the participants with only Time 1 and Time 2 data.

Time 1 assessment scores	Participants with Time 1 and 2 data only (*n* = 31)	Participants with Time 1, 2 and 3 data (*n* = 87)
	Mean (SD)	Mean (SD)
**LEAP vocabulary**	34.91 (12.59)	41.55 (8.85)
**LEAP ICW expressive language**	1.87 (1.80)	2.28 (2.09)
**LEAP ICW receptive language**	5.68 (2.95)	6.89 (2.49)
**RAPT grammar raw score**	7.69 (6.25)	13.13 (7.12)
**RAPT grammar percentile**	2.68 (5.28)	11.41 (18.97)
**RAPT information raw score**	13.38 (8.05)	19.38 (7.45)
**RAPT information percentile**	6.56 (11.65)	16.09 (16.92)

Table 5 shows the characteristics of the sub‐cohort with Time 3 data, by group (the intervention group who completed LEAP and the control group). There were no differences between the group who received LEAP and those who did not.

**TABLE 5:** Characteristics of Time 3 (follow‐up) sub‐cohort by group at Time 1.

		Intervention group (*n* = 41)	Control group (*n* = 46)
**Age in months *M* (SD)**		57.63 (6.29)	56.87 (5.99)
**Gender** **girls: boys (%)**		22 (53.7): 19 (46.3)	23 (50): 23 (50)
**EAL (%)**		11 (26.8)	10 (21.7)
**School year group**	**Reception (%)**	33 (80.5)	38 (80.5)
	**Year 1 (%)**	8 (19.5)	8 (17.4)

Table 6 shows the performance at Time 1, Time 2 and Time 3 by group for all five language measures, for participants who had complete data at Time 3. Analysis via two factor mixed design ANOVA indicated that there were no significant interactions between group (control vs. LEAP) and time (Time 1, Time 2 and Time 3) for LEAP vocabulary (*F*(2, 84) = 1.28, *p* = 2.85); LEAP ICW receptive language (*F*(2, 84) = 0.157, *p* = 0.855, partial eta squared 0.004); RAPT information score (*F*(2, 84) = 0.673, *p* = 0.513, partial eta squared 0.016); or RAPT grammar raw score (*F*(2, 84) = 1.49, *p* = 0.231, partial eta squared 0.034). There was a significant effect for LEAP ICW expressive language (*F*(2, 84) = 3.11, *p* = 0.05, partial eta squared 0.069).

**TABLE 6:** Performance at Time 1, Time 2 and Time 3 on outcome measures by group.

	Intervention group *N* = 41			Control group *N* = 46		
	Time 1	Time 2	Time 3	Time 1	Time 2	Time 3
	*M* (SD)	*M* (SD)	*M* (SD)	*M* (SD)	*M* (SD)	*M* (SD)
**LEAP vocabulary outcome measure**	41.10 (8.53)	45.07 (8.03)	45.46 (4.75)	41.96 (9.19)	44.52 (8.09)	45.20 (8.01)
**LEAP ICW expressive language**	2.00 (1.93)	4.29 (2.57)	4.16 (2.24)	2.52 (2.20)	3.43 (2.40)	4.09 (2.75)
**LEAP ICW receptive language**	6.85 (2.63)	7.78 (1.73)	7.83 (1.43)	6.91 (2.39)	7.63 (2.03)	7.59 (2.26)
**RAPT grammar raw score**	12.50 (7.22)	15.57 (6.74)	17.06 (6.87)	13. (7.06)	14.82 (6.39)	15.91 (6.92)
**RAPT grammar percentile**	7.50 (11.23)	15.32 (20.92)	18.15 (24.43)	14.80 (23.35)	13.07 (17.32)	15.37 (22.00)
**RAPT information raw score**	19.01 (8.12)	23.28 (8.49)	23.51 (6.79)	19.71 (6.96)	22.54 (8.13)	23.41 (7.83)
**RAPT information percentile**	13.74 (16.33)	28.31 (26.07)	27.45 (26.87)	18.17 (17.34)	28.98 (27.88)	30.13 (26.89)

## 5 Discussion

This study evaluated the impact of a language intervention programme that focused on using CSS with primary school aged children, differentiated into groups based on the ICW construct. The intervention—the Language Enrichment Activity Programme (LEAP)—was delivered twice weekly to small groups of children aged between 4 and 6 years for a school half term. This evaluation study compared the scores of children who completed LEAP (*n* = 70) and a waiting control (*n* = 48) on bespoke LEAP language assessment tasks and on the Renfrew Action Picture Test at two time points. A sub‐cohort was also followed up approximately 8 weeks after the LEAP finished (41 who received LEAP and 46 from the control group). To guard against expectancy bias, the trainee SLTs swapped schools and were blind as to whether the child had received LEAP or not. Results were promising: following the language groups, the children who completed the LEAP intervention had higher scores on the RAPT grammar raw score; the LEAP Vocabulary task, a non‐standardised assessment where children were asked to name 50 pictures; and the LEAP ICW Expressive, a non‐standardised assessment where children were asked to describe what was happening in a scene involving toys and objects, with answers scored according to how many ICW they included in their response. For the sub‐cohort assessed again approximately 8 weeks after LEAP ended, there was an interaction between time and group for the latter outcome measure only.

The study fits with evidence for implementation of targeted Tier 2 language intervention groups, typically with younger children in nursery and preschool settings (Clegg et al. 2020; Fricke et al. 2013; Lonigan and Phillips 2016; Reeves et al. 2018; Trebacz et al. 2024; Walker et al. 2020). LEAP offers a potentially effective support for language skills in early primary school years, an important period given the focus on early literacy instruction in the classroom (Snow 2016). Our findings are particularly encouraging given that the language groups were shorter in duration and intensity when compared to many such interventions: A systematic review found that Tier 2 intervention programmes typically worked with children 5 times weekly for a period of 11 weeks (Goldfeld et al. 2022), with LEAP involving two relatively short sessions per week over just 6 weeks. Our collaboration with trainee SLTs also made the programme very cost‐effective for schools (Snowling et al. 2022).

SLTs in the United Kingdom very commonly base interventions around the ICWs construct, where tasks involve children following structured, play based directions with variation in the amount and variety of words that carry meaning in a sentence, with children then asked to participate by describing play based scenarios with increasing potential ICWs in their responses (Morgan et al. 2019). Very little research has examined the ICW construct, despite its use since the 1970s (Masidlover 1979). There is currently very little research into the clinical use of the ICW construct to differentiate and structure language interventions, making our study an important contribution to discussions about the widespread application of the ICW construct by SLTs in the United Kingdom.

LEAP was designed by a team of speech and language therapists in Sheffield to meet the needs of children with language *difficulties* considered likely to respond to a period of intensive support within schools, rather than the specialist interventions needed for children with lifelong diagnoses. Despite calls for further implementation of such ‘tier two’ small group interventions, they can be challenging to implement, particularly given current high levels of school staff shortages and child absences (Dockrell et al. 2023). Our study worked with trainee SLTs to deliver LEAP in response to schools reporting high levels of unmet need. Our partnership had the dual benefit of increasing placement capacity for universities while also increasing the number of children who could receive language groups in our partner schools. Trainee SLTs have a wealth of background knowledge and expertise in child language development and use of CSS, which may be linked to greater success in the impact of the groups. For this project, they received four training workshops (8 h) and ongoing observation, feedback, peer support and tutor led clinical supervision throughout their delivery of the groups. While it was not a focus of our data collection, anecdotally students reported that they enjoyed delivering the intervention and that they developed transferable clinical skills (e.g., in session management, building rapport with children, and collaboration with school staff) and knowledge (e.g., around the evidence base for CSS, theories of word learning, and the information carrying word construct). Our project also opened up discussions about how clinicians use and contribute to evidence‐based practice in their work. The use of a delayed waiting control group fit well across two university semesters and could be a model for future collaborative implementation and evaluations of intervention programmes in schools.

Our results indicate that children made more progress on expressive language tasks within our outcome assessments than on receptive language tasks. There is an urgent need for research into how to go beyond compensating for children's current language processing skills towards *improving* comprehension skills (Tarvainen et al. 2021). It could be that participants’ increased scores on expressive rather than receptive measures indicated that this was the area where children made the most progress. However, it may also be due to the nature of our outcome measures. There were only three items on each of the LEAP ICW assessments, which may not have been enough to capture progress receptively. LEAP can be considered a complex intervention due to the number of language components involved in sessions; the range of behaviours targeted, covering both expressive and receptive language skills organised around the ICW construct; expertise and skills required by those delivering the intervention; and the permitted level of flexibility of the intervention sessions, around the manualised session plans (Skivington et al. 2021). Our outcome measures were driven by logistics such as administration time. Ideally, LEAP and similar intervention programmes should be evaluated using frameworks for developing and evaluating complex interventions, working from feasibility towards a better understanding of the impact of interventions, theorising how the intervention works and examining intervention components within the context of delivery (Skivington et al. 2021).

Although our findings are encouraging, they are preliminary given the multiple limitations to our design. The school closures of 2020/2021 led to a smaller waiting control group and missing data at Time 3 for 31 participants. There were significant differences between our groups at baseline, and the sub‐cohort who were followed up at Time 3 had higher scores at baseline than the overall cohort. Speculatively, this could be at least partly due to the impact of the pandemic on the language skills of some of those children or the impact of delivering LEAP with adults wearing face masks. Trainee SLTs were observed and received feedback on how they delivered LEAP to ensure fidelity to the programme, but we did not systematically collect fidelity data, and in fact we expected the LEAP sessions to have some variation as they were tailored for children's strengths, difficulties and interests. In addition, we did not have access to individual children's attendance data, and the number of sessions attended was not factored into our analysis. Importantly, our cohort size prevented examination of mediating factors that may impact the success of the intervention, such as EAL status, known language disorders, age or school attended. The children who participated in our study vary in their profiles, reflecting the translational and ‘real world’ nature of our project. Further research is needed to unpack who LEAP—and similar intervention programmes—is best targeted at.

We might expect the intervention to be of greatest benefit to children from socioeconomically disadvantaged backgrounds, based on previous research. For example, subgroup analysis of the NELI evaluation found pupils eligible for FSM who received the NELI programme made more progress than those not eligible for FSM (Smith et al. 2023). Although our partner schools were in areas associated with socioeconomic disadvantage, we did not examine whether LEAP was particularly beneficial to those children experiencing the most socioeconomic disadvantage.

Three of our eight partner schools serve communities where the majority of children are dual language learners. We did not want to exclude schools or children from participating, given that our broad aim was to facilitate more equal access to language support. We therefore included children regardless of EAL status, as have some previous evaluations of targeted language groups (West et al. 2021). This project was designed during the start of the most recent ‘decolonial turn’ in speech and language therapy, and with hindsight, there are important limitations in the design (Pillay et al. 2023). We were unable to work with bilingual co‐workers or interpreters during the project, due to practical limitations such as the variety of languages within the schools, for example, there would need to be multiple co‐workers per group. We also had a lack of locally available interpreters who speak Roma, which was a majority language in two of our schools. We aimed for the groups to celebrate and build upon children's skills across all languages spoken, but this aspect of the intervention was not a key focus and needs further research. We did not aim to diagnose children as having a language disorder as part of this project, and the language assessments were brief and indicative of need only. However, test administration and focus groups with EAL children in South Africa show that cultural and sociolinguistic factors influence responses to one of the assessments that we used, the Renfrew Action Picture test (Mdlalo et al. 2019). Following Freire (2000), Mdlalo et al. (2019) call for SLTs to be constantly self‐reflecting in how we engage with the challenge of EAL speakers, to avoid ‘turning a blind eye’ to issues (2231). The motivation for our project was to increase equitable access to support for language skills as children started primary school. Abrahams et al. (2019) suggest that population level clinical practice is one way that speech and language interventions can be delivered more equitably, for example, by working with a whole school of children rather than deciding eligibility using potentially biased and problematic criteria. Our project was designed with this ethos in mind, but we welcome future research and consideration of if and how targeted Tier 2 language groups can be part of creative, innovative approaches to addressing the needs of under‐served populations (Abrahams et al. 2019).

In conclusion, our structured language intervention—LEAP—was successfully delivered by trainee SLTs to children across eight schools, adding to evidence for the value of targeted interventions beyond children's pre‐school years. LEAP differentiated children according to whether they could understand and produce utterances of two, three or four ICW. Play‐based group work then gave language input and invitations to participate verbally at a level of ICW matched to a child's current level. CSS such as modelling, repetition, recasting, confirmation and use of gesture were used in each session. Children were supported to participate with scaffolded, flexible prompts so that learning was errorless. Our evaluation showed that completing LEAP led to gains in children's expressive language, as indicated by scores on bespoke outcome measures (expressive use of ICWs and a vocabulary checklist) as well as the grammar raw score on the RAPT. This finding is promising, and our study adds to an emerging evidence base supporting the application of the ICW construct within language interventions, a practice which is very common and long established as part of the Derbyshire Language Scheme (e.g., Masidlover 1979) and wider adapted approaches (Morgan et al. 2019) but which has only recently become the focus of research (Clegg et al. 2023; McKean et al. 2023). Further research is needed to investigate best practice in facilitating collaborative, equitable access to support for oral language skills during children's early primary school years.

## Data Availability

The data that support the findings of this study are available from the corresponding author upon reasonable request. The data are not publicly available due to privacy or ethical restrictions.
